# Clinicopathological features and EGFR gene mutation status in elderly patients with resected non–small-cell lung cancer

**DOI:** 10.1186/1471-2407-14-610

**Published:** 2014-08-25

**Authors:** Teppei Nishii, Tomoyuki Yokose, Yohei Miyagi, Yataro Daigo, Hiroyuki Ito, Tetsuya Isaka, Kentaro Imai, Shuji Murakami, Tetsuro Kondo, Haruhiro Saito, Fumihiro Oshita, Kouzo Yamada, Shoichi Matsukuma, Masahiro Tsuboi, Haruhiko Nakayama, Munetaka Masuda

**Affiliations:** Department of Thoracic Oncology, Kanagawa Cancer Center Hospital, 2-3-2 Nakao, Asahi-ku, Yokohama, 2418515 Japan; Department of Pathology, Kanagawa Cancer Center Hospital, 2-3-2 Nakao, Asahi-ku, Yokohama, 2418515 Japan; Molecular Pathology and Genetics Division, Kanagawa Cancer Center Research Institute, 2-3-2 Nakao, Asahi-ku, Yokohama, 2418515 Japan; Department of Medical Oncology and Cancer Center, Shiga University of Medical Science Hospital, Seta Tsukinowa-cho, Otsu, 5202192 Japan; Respiratory Disease Center, Yokohama City University Medical Center, 4-57 Urafune-cho, Minami-ku, Yokohama, 2320024 Japan; Department of Surgery, Yokohama City University Graduate School of Medicine, 3-9 Fukuura, Kanazawa-ku, Yokohama, 2360004 Japan

## Abstract

**Background:**

The rapid aging of the population in Japan has been accompanied by an increased rate of surgery for lung cancer among elderly patients. It is thus an urgent priority to map out a treatment strategy for elderly patients with primary lung cancer. Although surgical resection remains standard treatment for early stage non–small-cell lung cancer (NSCLC), it is now essential to confirm the status of epidermal growth factor receptor (EGFR) gene mutations when planning treatment strategies. Furthermore, several studies have reported that *EGFR* mutations are an independent prognostic marker in NSCLC. However, the relations between age group and the molecular and pathological characteristics of NSCLC remain unclear. We studied the status of *EGFR* mutations in elderly patients with NSCLC and examined the relations of *EGFR* mutations to clinicopathological factors and outcomes according to age group.

**Methods:**

A total of 388 consecutive patients with NSCLC who underwent complete tumor resection in our hospital from 2006 through 2008 were studied retrospectively. Formalin-fixed, paraffin-embedded tissue sections were used to isolate DNA from carcinoma lesions. Mutational analyses of EGFR gene exons 19, 20, and 21 and KRAS gene exons 12 and 13 were performed by loop-hybrid mobility shift assay, a highly sensitive polymerase chain reaction-based method.

**Results:**

*EGFR* mutations were detected in 185 (47.7%) and *KRAS* mutations were detected in 33 (8.5%) of the 388 patients. *EGFR* mutations were found in a significantly higher proportion of patients younger than 80 years (younger group; 178/359, 49.6%) than in patients 80 years or older (older group; 7/29, 24.1%) (P = 0.008). In contrast, *KRAS* mutations were more common in the older group (6/29, 20.7%) than in the younger group (27/359, 7.5%) (P = 0.014). The older group showed a trend toward a higher rate of 5-year overall survival among elderly patients with *EGFR* mutations (100%) than among those with wild-type *EGFR* (66.2%), but the difference was not significant.

**Conclusions:**

Our results suggest that the *EGFR* status of patients with NSCLC differs between patients 80 years or older and those younger than 80 years. *EGFR* mutation status might be a prognostic marker in elderly patients with completely resected NSCLC.

**Electronic supplementary material:**

The online version of this article (doi:10.1186/1471-2407-14-610) contains supplementary material, which is available to authorized users.

## Background

Primary lung cancer remains the leading cause of the death from malignant tumors worldwide [1]. Non–small-cell lung cancer (NSCLC) accounts for approximately 80% of all cases of lung cancer [2]. Although surgical resection remains the standard treatment for early NSCLC, several molecular pathways have been shown to have prognostic significance in NSCLC. The epidermal growth factor receptor (EGFR) pathway is considered particularly important. EGFR is a membrane glycoprotein with an extracellular ligand-binding domain, a transmembrane lipophilic segment, and an intracellular domain that has tyrosine kinase activity. When a growth factor binds to EGFR, EGFR is self-phosphorylated by tyrosine kinase, and phosphorylated EGFR activates cell-signaling pathway involved in the regulation of cell cycle, apoptosis, angiogenesis, and cellular proliferation. Specific mutations of *EGFR* induce constant phosphorylation of EGFR, and increased levels of phosphorylated EGFR activate downstream signals that induce carcinogenesis [3, 4]. *EGFR* mutations predict the effect of EGFR tyrosine kinase inhibitors (EGFR-TKI) [5, 6]. It is now essential to confirm *EGFR* mutation status when planning treatment strategies for advanced or recurrent NSCLC.

The population of Japan is aging rapidly. In 2011 the average life-span in Japan was 83 years (males 79 years, females 86 years) [7]. Aging of the population is accompanied by a rapid increase in the incidence of primary lung cancer as well as the number of operations for lung cancer among elderly patients. Since 2009 persons 80 years or older have accounted for more than 10% of all patients in Japan. In 2011, patients 80 years old or older accounted for 11.5% of all patients [8–12]. Aging will become a global problem in the future, and knowledge acquired in Japan may contribute to solving related problems. Previous studies have suggested a relation between *EGFR* mutations and several clinicopathological factors, but whether *EGFR* status differs according to age group remains unclear. The present study assessed the status of *EGFR* mutations in elderly patients with NSCLC and examined the relations of *EGFR* mutations and clinicopathological factors to outcomes.

## Methods

### Patients

We retrospectively studied 388 consecutive patients with NSCLC who underwent complete tumor resection at Kanagawa Cancer Center Hospital (Yokohama, Japan) from 2006 through 2008. This study was approved by the ethics committee of the Kanagawa Cancer Center, and informed consent was obtained from all patients. The pathological diagnoses were independently made by 2 pathologists (T.N., T.Y.). Discrepancies in diagnoses were resolved by mutual agreement. The median follow-up time was 1981 days.

### Assessments

Formalin-fixed, paraffin-embedded tissue sections of the resected tumors were used for DNA extraction. Mutational analyses of *EGFR* gene exons 19, 20, and 21 and *KRAS* gene exons 12 and 13 were performed by loop-hybrid mobility shift assay (LH-MSA), a highly sensitive polymerase chain reaction–based method, as described previously (Additional file 1: Table S1) [13].

### Statistical analysis

Relations between *EGFR* status and categorical data were evaluated with the chi-square test. Continuous variables were compared by Student’s t-test. Survival curves were plotted using the Kaplan-Meier method, and differences in survival rates were assessed using the log-rank test. P < 0.05 was considered to indicate statistical significance. Statistical manipulations were performed using the IBM SPSS Statistics 20 for Windows software system (IBM Corp, Armonk, NY, USA).

## Results

### Relations between *EGFR, KRAS*status and clinicopathological features

The patients’ characteristics are summarized in Table 1. Of the 388 patients, 228 (58.8%) were men, and 160 (41.2%) were women. The mean age was 66.6 years (range, 35–90). *EGFR* mutations were detected in 185 patients (185/388, 47.7%) and *KRAS* mutations were detected in 33 (33/388, 8.5%). *EGFR* mutations were found more frequently in women (110/185, 59.5%), adenocarcinoma (183/185, 98.9%), and non-smokers (106/185, 57.3%) (P < 0.001). Patients with *EGFR* mutation had fewer pre-existing cardiopulmonary comorbidities than patients with wild-type (P = 0.028). The mean tumor diameter was smaller in patients with *EGFR* mutations (2.68 ± 0.92 cm) than in those with wild-type *EGFR* (3.35 ± 1.71 cm; P < 0.001). The rate of pathological T1 disease was significantly higher among patients with *EGFR* mutations (114/185, 61.6%) than among those with wild-type *EGFR* (83/203, 40.9%; P < 0.001). In contrast, *KRAS* mutations were not significantly related to gender, histopathological type, or smoking status. Although *KRAS* status did not correlate with pathological T factors, mean tumor diameter was larger in patients with *KRAS* mutations (3.46 ± 1.99 cm) than in those with wild-type *KRAS* (2.99 ± 1.36 cm; P = 0.001).

**Table 1:** **Correlations between** ***EGFR*** **mutations and clinicopathological features**

Characteristics	Total	No. of patients					
		***EGFR***status		***p*** ^***a***^	***KRAS***status		***p*** ^***a***^
		Mutation	Wild-type		Mutation	Wild-type	
	(***n*** = 388)	(***n*** = 185, 47.7%)	(***n*** = 203, 52.3%)		(***n*** = 33, 8.5%)	(***n*** = 355, 91.5%)	
Mean age, yr ± SD^*b*^	66.6 ± 10.0	65.1 ± 10.3	67.9 ± 9.57	0.462	68.6 ± 9.11	66.4 ± 10.1	0.553
Gender				<0.001			0.552
Male	228	75	153		21	207	
Female	160	110	50		12	148	
Histological type				<0.001			0.059
Adenocarcinoma	302	183	119		30	272	
Others	86	2	84		3	83	
Vascular invasion							
Ly -	314	155	159	0.172	25	289	0.429
Ly +	74	30	44		8	66	
V -	261	151	110	<0.001	23	238	0.756
V +	127	34	93		10	117	
p-stage				<0.001			
I	293	155	138		22	271	0.217
II / III	95	30	65		11	84	
T-factor				<0.001			
T1	197	114	83		14	183	0.316
T2 / 3	191	71	120		19	191	
Tumor diameter (cm)	3.03 ± 1.43	2.68 ± 0.92	3.35 ± 1.71	<0.001	3.46 ± 1.99	2.99 ± 1.36	0.001
N-factor				0.348			
N0	322	157	165		29	293	0.435
N1 / 2	66	28	38		4	62	
Smoking status				<0.001			0.107
Non-smoker	157	106	51		9	148	
Smoker	231	79	152		24	207	
Pre-existing cardiopulmonary comorbidity	203	86	117	0.028	20	183	0.319

^a^p < 0.05 statistically significant.

^**b**^SD, standard deviation.

EGFR, epidermal growth factor receptor; KRAS, v-Ki-ras2 Kirsten rat sarcoma viral oncogene homolog; ND, lymph node dissection.

### Relations between age group and clinicopathological features

We divided the patients into two groups according to whether they were 80 years or older (older group) or younger than 80 years (younger group) and compared *EGFR* status and clinicopathological features between these age groups (Table 2). The younger group comprised 359 patients (92.5%), and the older group comprised 29 (7.5%). The proportion of patients with *EGFR* mutations was significantly higher in the younger group (178/359, 49.6%) than in the older group (7/29, 24.1%; P = 0.008). In contrast, *KRAS* mutations were more common in the older group (6/29, 20.7%) than in the younger group (27/359, 7.5%; P = 0.014). The proportion of smokers was significantly lower in the younger group (208/359, 57.9%) than in the older group (23/29, 79.3%; P = 0.024). Elderly patients had more pre-existing cardiopulmonary comorbidities than younger patients (P = 0.024). Gender, histopathological type, vascular invasion, pathological stage, and tumor diameter did not differ significantly between the groups. We omitted lymph-node resection in the older group (P < 0.001). Table 3 shows the region of *EGFR* mutation according to age group. Although the study group was small, there were no exon 20 mutations in the older group.

**Table 2:** **Correlations between age group and clinicopathological features, including** ***EGFR*** **status**

	No. of patients			
Characteristics	Total	≥80 years	<80 years	***p*** ^***a***^
	(***n*** = 388)	(***n*** = 29, 7.5%)	(***n*** = 359, 92.5%)	
Mean age, yr ± SD^*b*^	66.6 ± 10.0	82.6 ± 2.41	65.3 ± 9.29	<0.001
Gender				0.246
Male	228	20	208	
Female	160	9	151	
Histology				0.034
Adenocarcinoma	302	18	284	
others	86	11	75	
Biomarker				
*EGFR* wild type	203	22	181	0.008
*EGFR* mutation	185	7	178	
*KRA*S wild type	355	23	332	0.014
*KRAS* mutation	33	6	27	
Vascular invasion				
Ly -	314	26	288	0.214
Ly +	74	3	71	
V -	261	18	243	0.535
V +	127	11	116	
p-stage				0.080
I	293	18	275	
II / III	95	11	84	
T-factor				0.506
T1	197	13	184	
T2/3	191	16	175	
Tumor diameter (cm)	3.03 ± 1.43	3.00 ± 1.44	3.40 ± 1.24	0.629
N-factor				0.584
N0	322	23	299	
N1/2	66	6	60	
Operation				0.155
Limited resection (wedge/segmentectomy)	80	3	77	
Standard surgery (lobectomy, pneumonectomy)	308	26	282	
Lymph node resection				<0.001
ND0/1/sampling	109	25	156	
ND2	278	4	203	
Smoking				0.024
Non-smoker	157	6	151	
Smoker	231	23	208	
Pre-existing cardiopulmonary comorbidity	203	21	182	0.024

^a^p < 0.05 statistically significant.

^**b**^SD, standard deviation.

EGFR, epidermal growth factor receptor; KRAS, v-Ki-ras2 Kirsten rat sarcoma viral oncogene homolog; ND, lymph node dissection.

**Table 3:** **Region of** ***EGFR*** **mutation according to age group**

	No. of patients		
***EGFR***mutations	Total	≥80 years	<80 years
Exon 19	73	3	70
Exon 20	13	0	13
Exon 21	97	4	93
Combined	2	0	2

### Relations between *EGFR*status and outcomes

**Figure 1:**
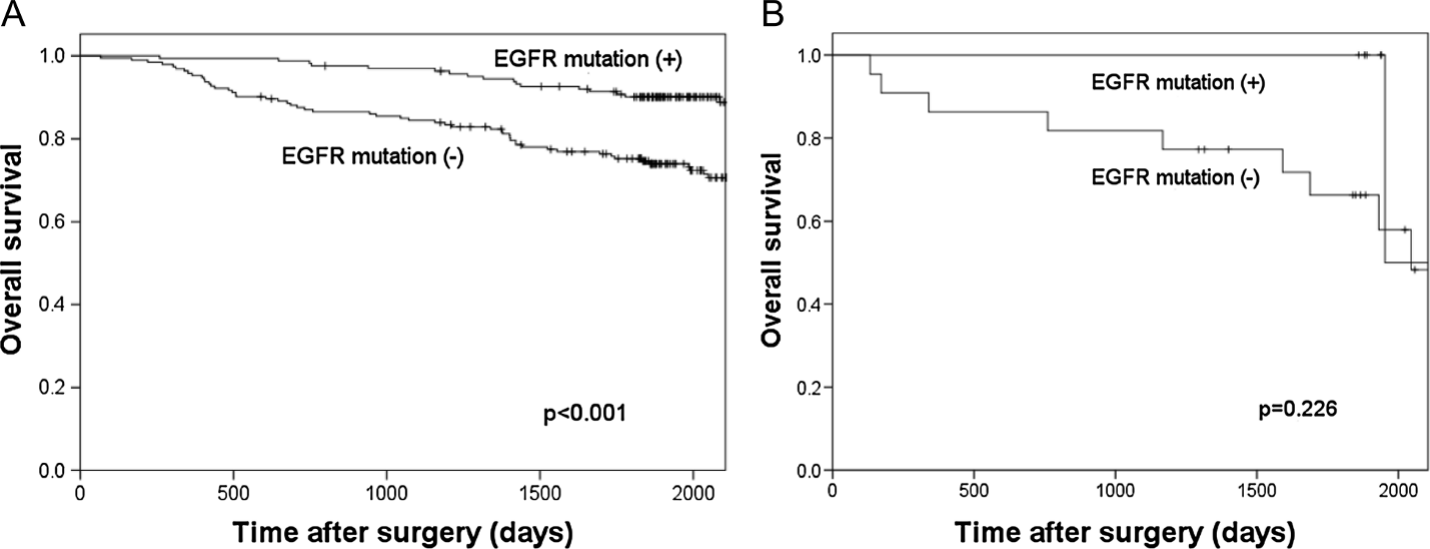
**Relations between** ***EGFR*** **mutations and outcomes.** Kaplan-Meier curve analysis showed that *EGFR* mutation status was significantly associated with survival. **(A)** The 5-year overall survival rate was higher in patients with *EGFR* mutations (90.2%) than in those with wild-type *EGFR* (75.2%) in the younger group (P < 0.001). **(B)** In the older group, the 5-year disease free survival rate was 100% among patients with *EGFR* mutations; however, the difference between the two groups was not significant.

Kaplan-Meier curve analysis showed that *EGFR* mutation status was significantly associated with survival (Figure 1). The 5-year overall survival rate was significantly higher in patients with *EGFR* mutations (90.2%) than in those with wild-type *EGFR* (75.2%) in the younger group (P < 0.001; Figure 1A). The 5-year overall survival rate was slightly, but not significantly higher in patients with *EGFR* mutations (100%) than in those with wild-type *EGFR* (66.2%) in the older group (P = 0.226; Figure 1B).

## Discussion

In the present study, we first evaluated *EGFR* mutations in resected NSCLC tissue by LH-MSA. LH-MSA is a highly sensitive polymerase chain reaction-based method. Sakuma *et al.* previously evaluated *EGFR* mutations by LH-MSA in our hospital. *EGFR* mutations were detected in 53.2% of NSCLCs and were significantly associated with adenocarcinoma, female sex, and no smoking history [14]. In the present study, we detected EGFR mutations in 47.7% of NSCLCs (Table 1). The presence of an *EGFR* mutation is closely linked to several clinicopathological factors, such as gender, smoking history, and pathological findings. Our results are consistent with those of recent studies reporting that the rate of EGFR mutations is higher among Asians (including Japanese), females, nonsmokers, and adenocarcinomas [14, 15]. Although LH-MSA yet has not been generally performed, it is known to be a sensitive and low cost method in scanning the known gene mutation. Furthermore, we can treat many samples in a short time by LH-MSA. Nakajima et al. analyzed *EGFR* mutations using LH-MSA, and confirmed the results by direct sequencing. They concluded that LH-MSA has a high detection capability compared with direct sequencing [16]. Guideline from the College of American Pathologists, International Association for the Study of Lung Cancer, and Association for Molecular Pathology indicate that LH-MSA compares favourably with the other method [17].

We then studied the relations between *EGFR* status and clinicopathological factors according to age group (Table 2). Past report suggested the impact of age on *EGFR* mutation, and concluded that age was associated with *EGFR* mutation in lung cancer [18]. In this study, if we analyze the *EGFR* status using the median age of 66 years old as a cutoff, there is no difference between younger and elderly group. Next, we divided the cohort in every ten years old, and we found that the rate of *EGFR* mutation suddenly decreased in a group 80 years or older. Because aging of the population is a global problem, the average life-span older than 80 years old in Japan was worthy of mention to the world. Due to the above reasons, we thought that the age of 80 years old is turning point in consideration of gene profile change, and divided the patients into two groups at 80 years of age. The older group (≥80 years) of patients with NSCLC included significantly higher rates of non-adenocarcinoma, wild-type *EGFR*, *KRAS* mutations, and smokers. There was no difference between the older group and younger group in tumor size, T-factor, or pathological stage. Moreover, in Japan, females outlive males (males 79 years, females 86 years). Of the 29 elderly patients, 9 are females include 7 adenocarcinomas and 4 smokers. *EGFR* mutations were detected in 3 females. The 5-year overall survival rate was 100% regardless of *EGFR* mutation or wild type. When we examined the region of *EGFR* mutation according to age group (Table 3), no exon 20 mutations were found in the older group. Although our study group was small, our results suggest that *EGFR* mutation status might differ between elderly and younger patients with NSCLCs. Given that smoking is one of the causes of the low rate of *EGFR* mutations in the older group, the rate of *EGFR* mutations may increase in the future owing to enlightenment movements such as the WHO Framework Convention on Tobacco Control [19]. Recently, smoking prevalence in Japan is decreasing generally. In particular, the drop of the smoking prevalence in young generation is remarkable. On the other hands, lung cancer mortality in Japan rises, probably it depends on the increase of the lung cancer in an elderly person who had been a smoker [20]. If the low rate of *EGFR* mutations is unrelated to smoking, it is very interesting that EGFR status might be affected by aging. Furthermore, it is reported that the response rate of gefitinib in elderly (aged 70 years or older) patients with advanced *EGFR* mutated NSCLC was 45.5%. EGFR-TKI is more effective than conventional chemotherapy in elderly patients, if we could pay attention to drug discontinuation and dose reduction due to age-related organ dysfunction [21]. On the other hand, NSCLC with exon 20 mutation is resistant for EGFR-TKI. Although our result has no statistical significance due to a small population of elderly patients, the lack of exon 20 mutations might be a characteristic of elderly patients. Large clinical trials are needed to investigate the relation between age group and the response to EGFR-TKI.

Finally, we assessed the relations between the *EGFR* status and outcomes. *EGFR* mutations were associated with significantly better survival than wild-type *EGFR* in the younger group (Figure 1). In the older group, however, the 5-year overall survival rate did not differ significantly according to *EGFR* mutations, and wild-type *EGFR* status and was 100% in patients with *EGFR* mutations. EGFR-TKIs are obviously beneficial in patients with advanced or recurrent NSCLC, but several studies have suggested that *EGFR* mutations might be an independent positive prognostic factor [22]. Our results suggest that elderly patients with NSCLC who have *EGFR* mutations are especially likely to have good outcomes after complete lung resection.

## Conclusion

Our results suggest that the *EGFR* status of patients with NSCLC differs according to age group (>80 years vs. ≤80 years). *EGFR* mutation status might be a prognostic marker in elderly patients with completely resected NSCLC.

## Electronic supplementary material

Additional file 1: Table S1: PCR Primers and LH-G Probes Used for Detection of Mutations in *EGFR*. (XLS 32 KB)
